# Optimization of an In Vitro Transcription/Translation System Based on *Sulfolobus solfataricus* Cell Lysate

**DOI:** 10.1155/2019/9848253

**Published:** 2019-02-11

**Authors:** Giada Lo Gullo, Rosanna Mattossovich, Giuseppe Perugino, Anna La Teana, Paola Londei, Dario Benelli

**Affiliations:** ^1^Department of Cellular Biotechnologies and Haematology, Sapienza University of Rome, Via Regina Elena 324, 00161 Rome, Italy; ^2^Institute of Biosciences and BioResources, National Research Council of Italy, Via Pietro Castellino 111, 80131 Naples, Italy; ^3^Department of Life and Environmental Science, Polytechnic University of Marche, Via Brecce Bianche, 60131 Ancona, Italy

## Abstract

A system is described which permits the efficient synthesis of proteins *in vitro* at high temperature. It is based on the use of an unfractionated cell lysate (S30) from *Sulfolobus solfataricus* previously well characterized in our laboratory for translation of pretranscribed mRNAs, and now adapted to perform coupled transcription and translation. The essential element in this expression system is a strong promoter derived from the *S. solfataricus* 16S/23S rRNA-encoding gene, from which specific mRNAs may be transcribed with high efficiency. The synthesis of two different proteins is reported, including the *S. solfataricus* DNA-alkylguanine-DNA-alkyl-transferase protein (*Ss*OGT), which is shown to be successfully labeled with appropriate fluorescent substrates and visualized in cell extracts. The simplicity of the experimental procedure and specific activity of the proteins offer a number of possibilities for the study of structure-function relationships of proteins.

## 1. Introduction

Cell-free protein synthesis (CFPS) systems have been used initially to investigate certain fundamental aspects of cell biology, such as deciphering the structure of the genetic code or elucidating the basic features of transcriptional and translational control [1–3]. Later, CFPS systems turned out to be also powerful tools to produce high amounts of proteins for a wide range of applications ranging from pharmaceutical use to protein structure analysis [4, 5].

The simplest forms of these systems consist of whole cell lysates (S30 extracts) containing all the necessary elements for transcription, translation, protein folding, and energy metabolism. Typically, CFPS systems are programmed for expression of proteins using two different substrates: RNA templates for translation only or DNA templates for coupled transcription/translation [6, 7].

The advantages of CFPS systems over *in vivo* methods are manifold. One can dispense with all the procedures required to support cell viability and growth; moreover, handling cellular extracts instead of whole cells facilitates the active monitoring, rapid sampling, and direct manipulation of the protein synthesis process. Last but not least, the simplicity and low cost of preparing cellular extracts make the system a preferential choice among the available tools for the synthesis of proteins of interest.

The most commonly used cell-free translation systems consist of *Escherichia coli* (ECE) extracts, rabbit reticulocytes (RRL), wheat germ (WGE), and insect cells (ICE), each of them with peculiar characteristics [8–10]. *E. coli* CFPS is the most convenient economically, since extract preparation is simple and inexpensive and the required proteins can be produced in high yields. However, CFPS derived from extracts of eukaryotic cells may be the best choice when the scope is the production of some types of complex proteins or when eukaryotic posttranslational modifications are required.

In our laboratory, we have developed a CFPS from the thermophilic archaeon *S. solfataricus*, which we have successfully used to decipher a number of aspects of archaeal translation and high-temperature translation[11, 12]. However, our standard system uses only pretranscribed RNA templates, while CFPS from hyperthermophiles allowing a coupled transcription/translation based exclusively on endogenous components of the adopted system have not so far, to the best of our knowledge, been described.

Yet, to develop such a system is highly desirable for a number of reasons. First of all, it represents a powerful tool to expand our understanding of the molecular mechanisms governing coupled transcription-translation in archaea. Moreover, the expression of recombinant proteins in thermophilic conditions similar to the native ones could facilitate the identification of associated factors. Furthermore, although mesophilic hosts such as *Escherichia coli* have been used to produce thermostable proteins for biochemical and crystallographic characterization [13], many hyperthermophilic proteins correctly fold only under physiological conditions of high temperature or in the presence of their native posttranslational modifications [14, 15].

We report here the development of a coupled *in vitro* transcription/translation system for cell-free protein synthesis from the thermophilic archaeon *S. solfataricus*. The system works with a plasmid vector obtained by cloning the strong promoter derived from the *S. solfataricus* 16S/23S rRNA-encoding gene upstream of a previously well-characterized *Sulfolobus* gene [16]. A preliminary assessment of the various parameters and components that affect the rate and yield of protein synthesis was performed. With this system, we obtained the *in vitro* expression of two different proteins, one of which was also shown to be enzymatically active at the temperature of 70°C.

## 2. Materials and Methods

### 2.1. Preparation of Cell Extracts and Total tRNA

Cell lysates competent for *in vitro* translation were prepared according to a method described previously with slight precautions [17]. Briefly, about 2 g of frozen cells were ground by hand with a double amount of alumina powder and adding gradually about less than 2 volumes (relative to the weight of the cell pellet) of lysis buffer (20 mM Tris-HCl (pH 7.4), 10 mM Mg(OAc)_2_, 40 mM NH_4_Cl, and 1 mM DTT). The procedure was performed by placing the mortar on ice and working in a cold room for no more than 15 min. Cell debris and alumina were removed by spinning the mix twice at 30,000 ×g for 30 min and taking care to withdraw only about two-thirds of the supernatant. Aliquots of the cell lysate (0.05 ml) were stored at −80°C, and total protein concentration, determined by Bradford assay, was in the range of about 20–25 mg/ml accordingly. Unfractionated tRNA from *S. solfataricus* was prepared by performing a phenol extraction of the crude S100 fraction and precipitating the aqueous phase with 2.5 volumes of 95% ethanol. The RNA pellet was resuspended in 10 mM glycine (pH 9.0), and the solution was incubated for 2 h at 37°C to achieve alkaline deacylation of the tRNA therein contained. Lastly, the RNA was again precipitated and the resulting pellet was dissolved in an adequate volume of 10 mM Tris-HCl (pH 7.5).

### 2.2. Gene Constructs and *In Vitro* Transcription

We used the plasmid pBluescript-SK(+) as a starting point for our subsequent constructs. Two synthetic DNA oligomers of 48 nucleotides were designed on the sequence of a 16S/23S rRNA operon promoter described elsewhere [18] whose sequence is identically conserved in all *S. solfataricus* species: promoter rRNA SSO forward 5′-CGAAGTTAGATTTATATGGGATTTCAGAACAATATGTATAATGGGTAC-3′ and promoter rRNA SSO reverse 5′-CCATTATACATATTGTTCTGAAATCCCATATAAATCTAACTTCGGTAC-3′. Both primers contained at their 5′ ends a sequence corresponding to the protruding cohesive 5′ end of the *Kpn* I restriction site, and both were phosphorylated in separate 25 *μ*l reaction mixtures containing 70 mM Tris-HCl (pH 7.6), 10 mM MgCl_2_, 5 mM dithiothreitol, 1 mM ATP, 4 *μ*M DNA, and 10 units of T4 polynucleotide kinase (New England BioLabs). After incubation at 37°C for 1 h, the reaction mixtures were combined and the kinase was heat-inactivated at 70°C for 10 min. Annealing of the two oligomers was obtained by heating this mixture at 100°C for 4 min and slowly cooling down to 37°C. The integrity of the double-stranded 16S/23S rRNA promoter fragment DNA was checked by agarose gel electrophoresis. One pmol of the purified double-stranded fragment was incubated with 0.25 pmol of *Kpn* I digested pBS-SK(+) plasmid in the presence of 10 units of T4 DNA ligase (New England BioLabs) in 25 *μ*l of 50 mM Tris-HCl (pH 7.5), 10 mM MgCl_2_, 10 mM dithiothreitol, 1 mM ATP, and 25 *μ*g/ml bovine serum albumin for 20 h at 16°C. One-tenth of this reaction mixture was then used directly for transformation of *E. coli* top 10 competent cells. Transformants harbouring plasmid DNA were screened for the presence of the insert using a *Kpn* I restriction analysis of purified plasmid DNA. The clone harbouring the construct with the insert in the correct orientation was selected after DNA sequencing and termed pBS-rRNA_p_. Successively, a fragment of 393 bp containing the gene termed ORF 104 with its Shine-Dalgarno (SD) motif was amplified from the construct pBS800 [12] by PCR using the following primers: Prom-104 *Xho* I 5′-TTTTTTTATCTCGAGCCGGAATAGTTGAATTAACAATGAAGC-3′ (underlined sequence corresponds to the *Xho* I site) and Prom-104 *Pst* I 5′-CATGGTATGCTGCAGTCATTGCTTCACCTCTTTAATAAACTCC-3′ (underlined sequence corresponds to the *Pst* I site). The fragment was inserted into the *Xho* I*-Pst* I digested plasmid pBS-rRNA_p_, yielding the construct termed pBS-rRNA_p_-104. To generate the construct termed pBS-rRNA_p_-*ogt*, we excised the fragment *Xho* I*-Pst* I from the previous plasmid and inserted a DNA fragment of 533 bp amplified from the construct pQE-*ogt* by PCR with the following primers: forward rRNA/*Ss*OGT *Xho* I 5′-TTTTTCTCGAGTGAGGTGAAATGTAAATGAGAGGATCTCACCATCACC-3′ (underlined sequence corresponds to the *Xho* I site) and reverse rRNA/*Ss*OGT *Pst* I 5′-TTTTTTCTGCAGTCATTCTGGTATTTTGACTCCC-3′ (underlined sequence corresponds to the *Pst* I site). Also in this case, the plasmid was designed to have the SD motif 7 nucleotides upstream of the *ogt* start codon.

### 2.3. Analysis of Transcriptional Activity of *Sulfolobus solfataricus* Lysate by *In Vitro* Labelling with ^32^P-UTP

The transcriptional activity of the *S. solfataricus* cell-free extract was tested by ^32^P-UTP incorporation in two different reaction conditions using an aliquot of the lysate corresponding to 100 *μ*g of total proteins. The first reaction protocol was adopted from a previous study [16]: the cell-free extract was incubated in a reaction volume of 50 *μ*l, in the presence of 50 mM Tris-HCl (pH 8.0), 25 mM MgCl_2_, 1 mM EDTA, 1 mM dithiothreitol, 2 mM ATP, 1 mM GTP, 1 mM CTP, 0.6 mM UTP, and 100 *μ*M (*α*-^32^P) UTP (4 Ci/mmol) in a reaction volume of 50 *μ*l. The reaction was carried out at 60°C for 30 min. The second protocol was based on the *in vitro* translation experiments carried out in our laboratory [12, 17]: *S. solfataricus* cell-free extract was incubated in a reaction volume of 50 *μ*l, in the presence of 10 mM KCl, 20 mM Tris-HCl (pH 6.8), 20 mM Mg(OAc)_2_, 2 mM ATP, 1 mM CTP, 1 mM GTP, 0.5 mM UTP, and 100 *μ*M (*α*-^32^P) UTP (4 Ci/mmol). The reaction, in this case, was carried out at 70°C for 30 min. At the end of both reactions, 20 U of DNase I were added and incubation was extended for 30 min at 37°C. DNase I was added to remove any trace of plasmidic DNA that could alter the results of the next qRT-PCR analysis. The products of the reactions were extracted by phenol pH 4.7 and precipitated with 2.5 volumes of 95% ethanol. The pellets were resuspended in an adequate volume of DEPC-treated water and divided into two aliquots. RNase A (20 *μ*g) was added to one of them and both aliquots were incubated at 37°C for 30 min. The newly synthesized RNA was separated by 8.5% of nondenaturing polyacrylamide gels and detected using both an Instant Imager apparatus (Packard) and autoradiography film (Kodak XAR-5).

### 2.4. *In Vitro* Translation and Coupled *In Vitro* Transcription-Translation

The transcription-translation activity was measured in a final volume of 25 *μ*l and contained 10 mM KCl, 20 mM Tris-HCl (pH 6.8), 20 mM Mg(OAc)_2_, 1.5 mM ATP, 1.5 mM CTP, 1.5 mM GTP, 1.5 mM UTP, 3.3 *μ*g of bulk *S. solfataricus* tRNA, 5 *μ*l of 20–25 mg/ml *S. solfataricus* S30 extract (preincubated for 10 min at 70°C), and 0.5 *μ*l of L-(^35^S)-methionine (S.A. 1175 Ci mmol^−1^ at 11 mCi ml^−1^, PerkinElmer). After mixing all components, 4 *μ*g of the desired mRNA or different amounts of the various plasmids were added and the mixtures were incubated for the indicated time at 70°C. Whole cell lysates were programmed for *in vitro* translation with transcripts of *S. solfataricus* genes ORF 104 and *Ss*OGT cloned in the pBS-SK(+) plasmid downstream of the T7 RNA polymerase promoter under conditions described in Table 1. Before transcription, the plasmids were linearized with *Pst* I. The experimental conditions were the same as described above except for the absence of CTP and UTP and the presence of ATP and GTP to the final concentration of 1.8 and 0.9 mM, respectively. The analysis of the translation products was performed by loading 15 *μ*l of the incubation mixture in 16% polyacrilamide/SDS gels; after the run, the gels were dried and autoradiographed.

### 2.5. qPCR and RT-PCR *Ss*OGT Labelling

At the end of *in vitro* transcription or coupled *in vitro* transcription-translation, total RNA was purified from the reactions by phenol extraction at pH 4.7 and precipitated by adding of 2.5 volumes of 95% ethanol. The pellets were resuspended in an adequate volume of DEPC-treated water and treated with 2 U of DNase I, RNase-free (Thermo Fisher Scientific) in an appropriate buffer at 37°C for 45 min. The residual products were reextracted by phenol pH 4.7 and precipitated with 2.5 volumes of 95% ethanol. 0.5 *μ*g of total RNA was retrotranscribed for relative qRT-PCR analysis (SensiFAST™ cDNA Synthesis Kit, Bioline). qPCR was performed with the Applied Biosystem StepOne Real-Time PCR System (Thermo Fisher Scientific) using 1/20 of cDNA and 10 *μ*l of GoTaq® qPCR Master Mix (Promega) in a final volume of 20 *μ*l. Cycling parameters were 95°C for 2 min, followed by 40 cycles of denaturation at 95°C for 3 sec, and annealing/extension at 60°C for 30 sec. The relative amount of each mRNA was calculated by the 2^−ΔΔCt^ method and normalized to endogenous aIF6 mRNA. Primer sequences used for qPCR were as follows: forward pBS 5′-TGGTAACAGGATTAGCAGAG-3′ and reverse pBS 5′-ACCAAATACTGTCCTTCTAGTG-3′; aIF6 forward 5′-ATAAGCGGTAACGATAACGG-3′ and aIF6 reverse 5′-AATCCCTTAGATTCTCCTTCAG-3′.

By performing RT-qPCR as described above, we measured the absolute amount of RNA transcribed from the plasmid pBS-rRNA_p_-104 following incubation in the in vitro transcription-translation system. Specifically, we compared the Ct values obtained from these samples with a standard curve plotted with Ct values obtained from serial dilutions of 1 *μ*g of in vitro transcribed RNA (pBS-rRNA_p_-104). For semiquantitative RT-PCR, total RNA was extracted from the mix reaction as described above. 2 *μ*g of total RNA was retrotranscribed in a final volume of 25 *μ*l with 200 U M-MLV reverse transcriptase in 20 *μ*l of mixture reaction for 1 h at 42°C according to the instructions of the supplier (Promega). The reaction contained 1 *μ*M of the following reverse primer: 5′-GGTTTCCCGACTGGAAAGCGGGCAG-3′. At the end of the reaction, the final volume of the mixture reaction was adjusted to 50 *μ*l and one-tenth of the RT reaction was PCR amplified with Taq DNA polymerase (Promega) for 30 sec at 95°C, 30 sec at 60°C, and 45 sec at 74°C (25 cycles) with a final extension step for 7 min at 74°C. Reverse primers for PCR amplification were the same used in the RT reaction coupled with the following forward primer: 5′-CGAATTCCTGCAGCCCGGGGGATCC-3′. The products of the reactions were separated by agarose gel electrophoresis and detected by ethidium bromide staining.

Controls correspond to reactions performed on RNA purified from samples in the absence of the plasmid and from RT minus cDNA reactions.

### 2.6. *Ss*OGT *In Vitro* Labeling

The activity of *in vitro*-expressed *Ss*OGT was analysed incubating 8 *μ*g of pBS-rRNA_p_-*ogt* plasmid or 200 ng of recombinant *Ss*OGT OGT with 200 *μ*g of *S. solfataricus* whole cell extract under the experimental conditions described above for coupled in vitro transcription/translation and in the presence of BG-FL substrate (2.5 *μ*M). The mix reaction was incubated at 70°C for 60 min. Reactions were stopped by denaturation, and samples were subjected to SDS-PAGE, followed by fluorescence imaging analysis using a VersaDoc 4000™ system (Bio-Rad Laboratories Inc.) by applying as excitation/emission parameters a blue LED bandpass filter. For western blot analysis, proteins were transferred onto PVDF filters (Bio-Rad Laboratories Inc.) using the Trans-Blot® Turbo™ Blotting System (Bio-Rad Laboratories Inc.). The presence of *Ss*OGT protein was revealed using polyclonal antibodies raised in rabbit against *S. solfataricus* OGT as primary antibodies, the goat anti-rabbit IgG-HRP (Pierce) as secondary antibody, and the Amersham Biosciences ECL Plus kit. Filters were incubated, washed, and developed according to the manufacturer's instructions. Chemiluminescent bands were revealed using a VersaDoc apparatus (Bio-Rad Laboratories Inc.).

## 3. Results and Discussion

### 3.1. Analysis of *In Vitro* Transcription in the S30 Fraction of *S. solfataricus*

**Figure 1:**
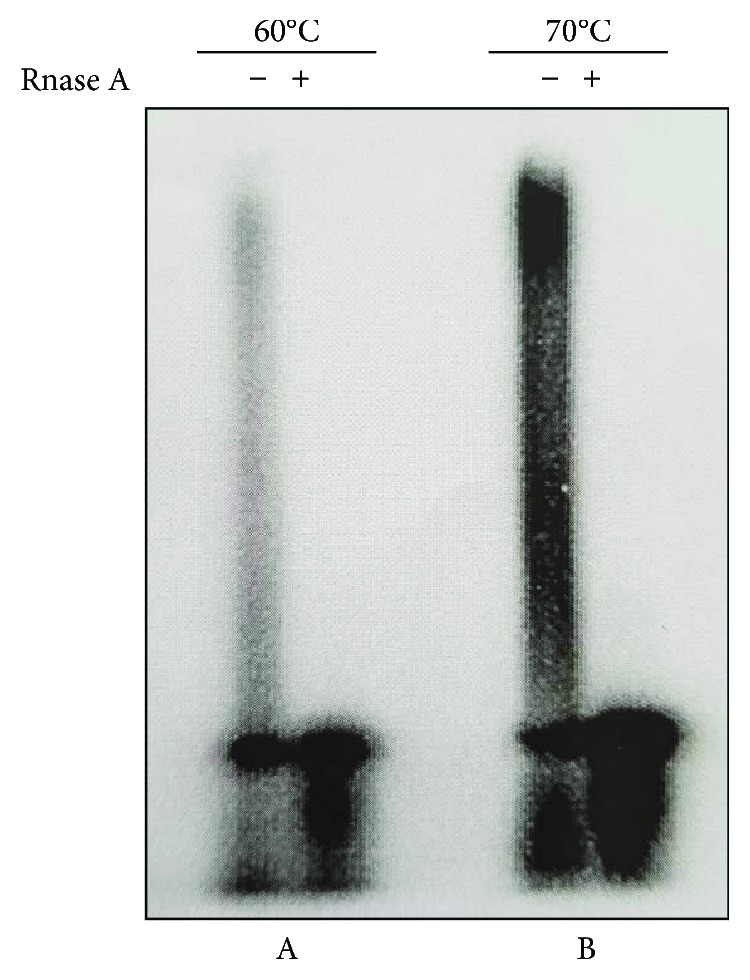
Transcriptional activity of *S. solfataricus* whole cell extracts. In vitro transcription reactions were performed using *S. solfataricus* S30 fractions with (*α*-^32^P) UTP in different experimental conditions as described in Material and Methods and Table 1. Reaction A was incubated at 60°C while reaction B was incubated at 70°C. Total RNA was extracted from the reaction mixes, and an aliquot of the samples was treated with RNase A at 37°C for 30 min. The products of in vitro transcription were subjected to nondenaturing polyacrylamide gel electrophoresis, and those incorporating (*α*-^32^P) UTP were visualized by autoradiography.

To prepare an S30 extract capable of efficient coupled transcription-translation, we performed preliminary experiments to verify whether the whole cell lysate of *S. solfataricus* prepared according to our described protocols [17] was competent for *in vitro* transcription. Specifically, we compared the transcriptional activity of our system with that of a previously described *Sulfolobus* in vitro transcription assay [16], testing the capacity of the S30 extract to incorporate *α*-^32^P-UTP. Salt and temperature conditions of the reactions are summarized in Table 1 and described in detail in Materials and Methods. In both cases, we implemented the reactions with the nucleoside triphosphates at the final concentration of 1 mM each (except ATP to 2 mM) and the S30 fraction was prepared omitting DNase I treatment of lysate, unlike previously published protocols where DNase I was added to remove genomic DNA [17]. As shown in Figure 1, both S30 extracts showed the ability to recruit labeled uridine triphosphate supporting the idea that endogenous RNA polymerase was active. However, the extract prepared according to our protocol had a higher efficiency of uridine triphosphate incorporation.

**Figure 2:**
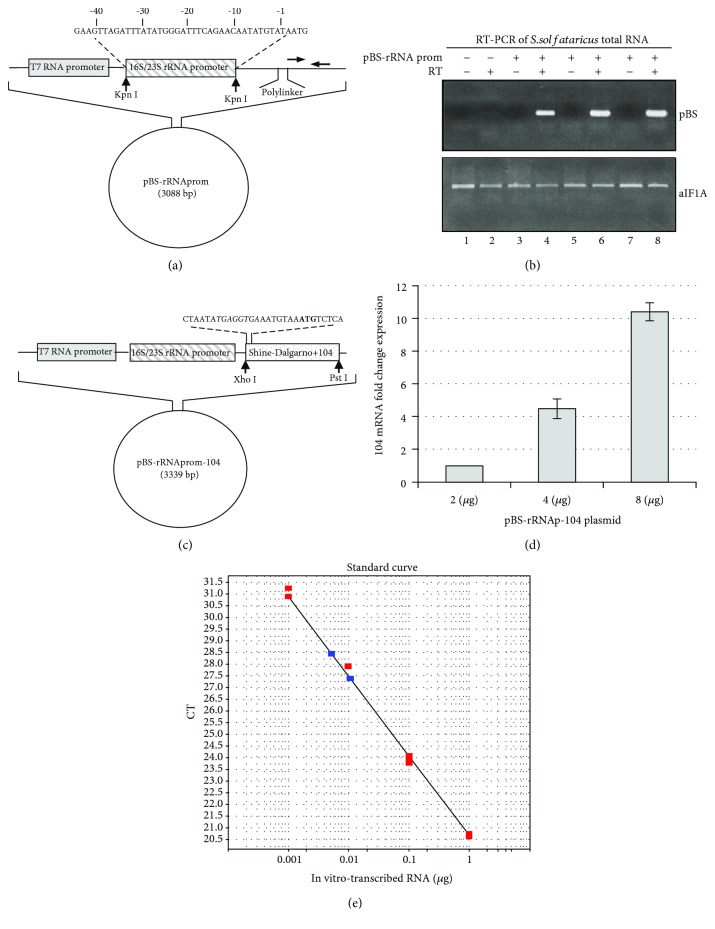
In vitro transcription of plasmids containing the 16S/23S rRNA promoter. (a) Schematic representation of the pBS-rRNA_p_ construct. Horizontal arrows indicate the position of primers used for RT-PCR analysis. (b) RT-PCR on total RNA extracted from S30 of *S. solfataricus* previously incubated with 2, 4 and 8 *μ*g of pBS-rRNA_p_ plasmid and corresponding, respectively, to the lanes 4, 6 and 8 of the image. The product of the reaction is shown by the amplified fragment of 346 bp. Also shown is the RT-PCR of an mRNA encoding the translation factor aIF1A, used as an endogenous control to normalize the reactions. (c) Schematic representation of the pBS-rRNA_p_-104 plasmid. The SD motif is evidenced in italic, while the start codon is shown in bold. (d) Relative amount of RNA transcribed by the pBS-rRNA_p_-104 plasmid incubated into *S. solfataricus* S30 extract at 70°C for 1 h. (e) Absolute quantification of the pBS-rRNA_p_-104 transcript using the standard curve method. The absolute quantities of the standards were obtained measuring the concentration of T7 in vitro-transcribed pBS-rRNA_p_-104 RNA. Serial dilutions of the in vitro transcript were obtained and their Ct values (red dots) were compared to those unknown (blue dots) extrapolating the amount of copies expressed.

Successively, based on a study characterizing the promoter for the single-copy 16S/23S rRNA gene cluster of the extremely thermophilic archaebacterium *Sulfolobus* [18], we cloned this promoter into the pBS-SK(+) plasmid, as described in Materials and Methods. The construct contained the region of DNA upstream from the transcription start site of the 16S/23S rDNA gene spanning from −1 to −40 bp. The structure of the construct, termed pBS-rRNA_p_, is shown schematically in Figure 2(a). The plasmid was incubated with the S30 extract, and its transcription was analysed by RT-PCR using primers annealing to a specific region of the plasmid downstream of the cloned gene, thus excluding amplification of the endogenous target. The results showed an efficient transcription of the plasmid following incubation at 70°C (Figure 2(b)).

Starting from this construct, we cloned a previously well-characterized *Sulfolobus* gene encoding a putative ribosomal protein [12], under the transcriptional control of the 16S/23S rDNA promoter. The structure of this plasmid, termed pBS-rRNA_p_-104, is shown schematically in Figure 2(c); analysis by qPCR showed that it was also transcribed (Figure 2(d)). Finally, the pBS-rRNA_p_-104 construct was transcribed in vitro with T7 RNA polymerase, and known amounts of the corresponding purified RNA were used to draw a calibration curve, which was used to quantify the transcription reactions (Figure 2(e)). This analysis permitted us to assess the amount of *in vitro*-transcribed RNA to an order of magnitude corresponding to ng of RNA for *μ*g of plasmid used, in 25 *μ*l of reaction.

## 4. Optimization of *In Vitro* Translation Conditions with respect to NTPs and Mg^++^ Ions

**Figure 3:**
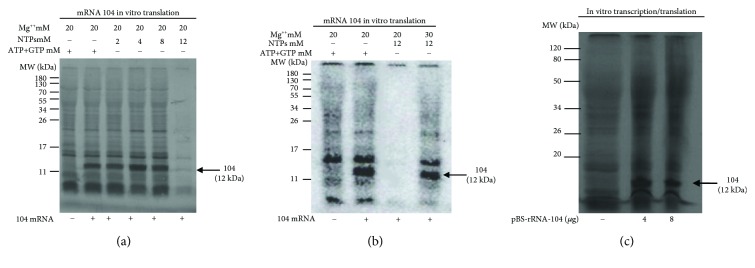
In vitro expression of ORF 104 under different experimental conditions. 4 *μ*g of in vitro-transcribed 104 mRNA were translated at different concentrations of NTPs (a) and Mg^2+^ (b) for 1 h in 25 *μ*l of reaction. (c) Different amounts of pBS-rRNA_p_-104 plasmid were incubated with *S. solfataricus* whole cell extract for 60 min at 70°C in a final volume of 25 *μ*l.

Next, we investigated whether the conditions adopted for *in vitro* transcription with the *S. solfataricus* S30 extract could affect its translational activity. Specifically, we sought to define an optimal concentration of NTPs since it is well known that free nucleotides chelate a proportional number of Mg^++^ ions, whose presence in a well-defined range of concentration is essential for translation [19]. For this purpose, we incubated the S30 extract with pretranscribed 104 mRNA in the absence or presence of different concentrations of NTPs and determined its translational efficiency. Indeed, increased levels of NTP in the mix reactions were detrimental for in vitro translation (Figure 3(a)). However, this could be in part compensated by increasing the concentration of Mg^++^ ions as shown in Figure 3(b). On the other hand, the absence of NTPs in the mix reaction completely inhibited the activity of the system, since exogenous ATP and GTP are required as an energy source (data not shown). Overall, based on the results of Figures 3(a) and 3(b), we chose to strike a balance between NTP and Mg^++^, setting them at the final concentration of 6 and 20 mM, respectively.

### 4.1. Transcription and Translation-Coupled Protein Synthesis

We then proceeded to verify whether the previously established experimental conditions allowed coupled transcription and translation. This question was addressed by incubating different amounts of the pBS-rRNA_p_-104 plasmid with the lysate at 70°C for 1 h under the conditions summarized in Table 1. As said before, the transcription of this construct from a strong rRNA promoter was expected to yield an mRNA encoding a ribosomal protein (ORF 104). The predicted mRNA was endowed with a 5′-UTR containing SD motif 7 nucleotides upstream from the AUG start codon of ORF 104. As shown in Figure 3(c), the reaction yielded a main protein band of about 12 kDa, corresponding to the expected size of the ORF 104.

To extend the above results to other *S. solfataricus* genes, we subcloned the *O*^6^-DNA-alkyl-guanine-DNA-alkyl-transferase gene (*Ss*OGT) from the pQE-*ogt* construct, previously characterized by Perugino et al. [20]. The product of this gene is a ubiquitous protein of about 17 kDa, evolutionary involved in the direct repair of DNA lesions caused by the alkylating agents. *Ss*OGT is a peculiar protein for its suicidal catalytic reaction: the protein irreversibly transfers the alkyl group from the DNA to a catalytic cysteine in its active site. The use of fluorescent derivatives of a strong inhibitor, the *O*^6^-benzyl-guanine (*O*^6^-BG), leads to an irreversible fluoresceinated form of this protein. This thermophilic variant of the so-called SNAP-tag™ [21] represents an alternative to the classical GFP-based systems and is eligible for our choice.

**Figure 4:**
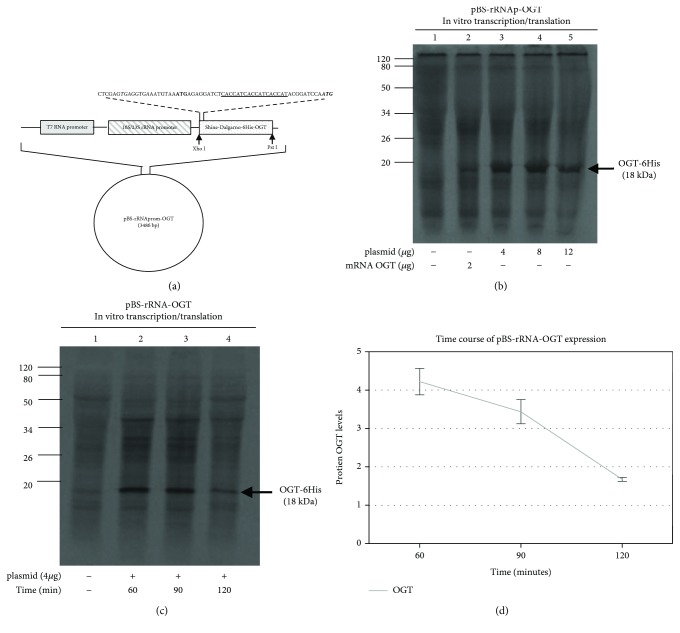
In vitro expression of OGT. (a) Schematic representation of the pBS-rRNA_p_-*ogt* plasmid. It was designed by introducing a DNA fragment of 522 bp containing the *ogt* gene into the *Xho* I-*Pst* I sites replacing ORF 104. The coding region starts with an AUG codon (bold letters) preceding a DNA region coding for six histidines (underlined letters) placed to the amino terminal region of the OGT protein (bold and italic letters). The DNA insert contains an SD motif (italic letters) retained from the ORF 104 and located 7 nucleotides upstream from the coding region. (b) Increased amounts of the pBS-rRNA_p_-*ogt* plasmid were incubated with *S. solfataricus* whole cell extract for 60 min at 70°C in a final volume of 25 *μ*l, and the products of expression were resolved by 16% denaturing polyacrylamide gel electrophoresis. (c) Time course of OGT expression: 4 *μ*g of pBS-rRNA_p_-*ogt* plasmid were incubated with *S. solfataricus* whole cell extract at 70°C and equal aliquots of the reaction were withdrawn from the mixture at the indicated times. (d) A graph is plotted with the values of the band intensity corresponding to the OGT protein shown in (c) and quantified using ImageJ software (NIH). The values represent the average of three independent experiments. All error bars indicate SD.

The construct was obtained by substituting the gene 104 from the construct pBS-rRNA-104 with the *ogt* gene, as described in Materials and Methods. The structure of the construct termed pBS-rRNA_p_-*ogt* is shown schematically in Figure 4(a).

Specifically, the strong SD motif 7 nucleotides upstream from the AUG start codon were retained, and 6 His-coding triplets were placed upstream of the *ogt* open reading frame. As the results in Figure 4(b) show, the gene was expressed producing a main protein band of about 18 kDa, corresponding to the expected size of the ORF *Ss*OGT-6His. As a positive control, we employed an *ogt* mRNA transcribed *in vitro* from the T7 promoter (lane 2), which, as expected, was translated less efficiently than the mRNA directly transcribed in the reaction mix. This is possibly due to the different 5′-UTR of the two mRNAs, but it is also conceivable that when translation takes place at the same time as transcription the mRNA is stabilized and the ribosomes may bind more easily to the translation start sites.

To gain insight into other factors influencing the efficiency of *Ss*OGT protein expression, we analysed the time course of the reaction with a fixed amount of the same construct. The highest expression level of the protein was observed after 60 min incubation, while at longer times (90 and 120 min) the efficiency decreased (Figures 4(c) and 4(d)), as observed in other *in vitro* expression systems [22]. This effect is probably due to the shortage of low molecular weight substrates (ATP, GTP, and amino acids) that are continuously used by the system with consequent drop of the reaction.

**Figure 5:**
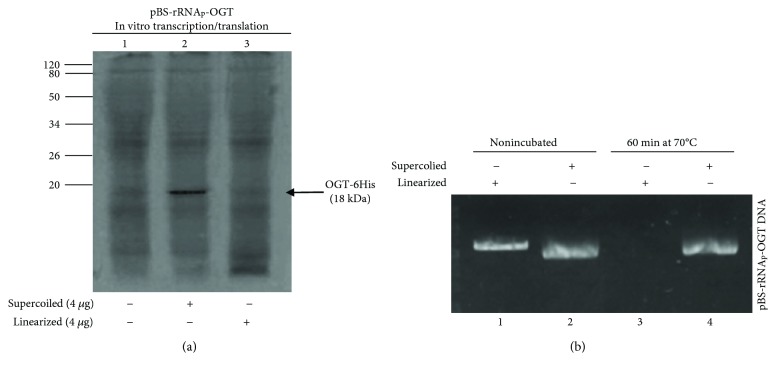
In vitro expression of *ogt* from linearized plasmid. (a) Supercoiled and linear pBS-rRNA_p_-*ogt* plasmids were incubated with *S. solfataricus* whole cell extract for 60 min at 70°C with ^35^S-Met in a final volume of 25 *μ*l, and the products of expression were resolved by 16% denaturing polyacrylamide gel electrophoresis. (b) Survival of supercoiled and linear pBS-rRNA_p_-*ogt* plasmid after incubation in the S30-coupled system. The constructs were incubated for 60 min at 70°C under standard conditions and then analysed on a 1% agarose gel. Lane 1, nonincubated linear pBS-rRNA_p_-*ogt* DNA; lane 2, nonincubated supercoiled pBS-rRNA_p_-*ogt* DNA; lane 3, linear pBS-rRNA_p_-*ogt* DNA incubated in an S30 mixture; and lane 4, supercoiled pBS-rRNA_p_-*ogt* DNA incubated in an S30 mixture.

Furthermore, we tested whether the linearization of the construct could produce a transcriptional runoff at the end of the gene with a consequent increase of the product of our interest. This was not the case, however. Samples incubated with the linearized plasmid failed to yield a band corresponding to the expected size of the ORF OGT-6His (Figure 5(a)). Further analysis revealed that this was due to degradation of the linearized plasmid in the reaction mix (Figure 5(b)) similar to results obtained by other authors with different cell-free coupled transcription-translation systems [23].

### 4.2. Characterization of *Ss*OGT Activity

**Figure 6:**
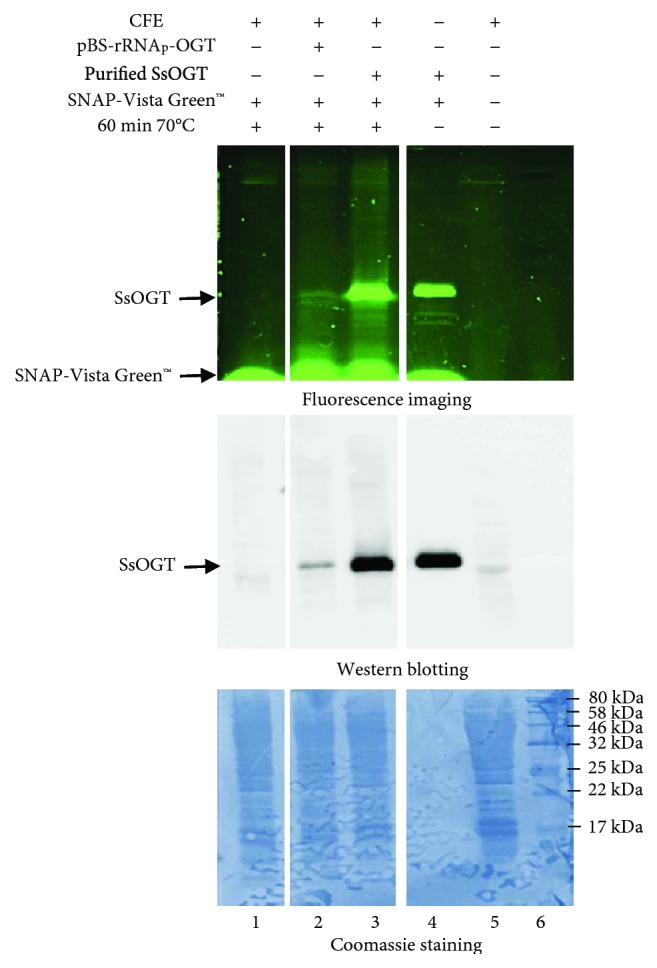
*Ss*OGT labeling. SDS-PAGE of in vitro-expressed pBS-rRNA_p_-*ogt* plasmid and purified *Ss*OGT protein both incubated with the BG-FL substrate (5 *μ*M) for 60 min at 70°C. The gel was exposed for fluorescence imaging analysis, blotted, and stained with Coomassie blue. The filter was probed with the anti-OGT antibody (middle panel). Lane 1 contains 100 *μ*g of *S. solfataricus* S30 fraction in the presence of the BG-FL substrate; lane 2 contains 8 *μ*g of pBS-rRNA_p_-*ogt* plasmid in 100 *μ*g of *S. solfataricus* S30 fraction and BG-FL substrate; lane 3 contains 200 ng of purified OGT protein with 100 *μ*g of *S. solfataricus* S30 fraction and BG-FL substrate; lane 4 contains 200 ng of purified OGT protein with BG-FL substrate; lane 5 contains 100 *μ*g of *S. solfataricus* S30 fraction; and lane 6 corresponds to the protein marker.

To test whether the *in vitro*-produced *Ss*OGT was functionally active, we incubated the construct pBS-rRNA_p_-*ogt* with the lysate at 70°C for 1 h in the presence of a fluorescein derivative of the *O*^6^-BG (SNAP-Vista Green™, New England BioLabs). As mentioned above, *Ss*OGT catalyzes the formation of a covalent bond between the benzyl group of BG and a specific cysteine residue in its active site; therefore, the successful completion of the reaction renders the protein fluorescent [21]. Indeed, we observed a fluorescent band corresponding to the expected size of the *Ss*OGT in the reaction conditions adopted (Figure 6), demonstrating the active state of the expressed protein. The levels of *in vitro*-expressed *Ss*OGT were assessed by comparing its fluorescence with that obtained with known amounts of recombinant protein. The outcome of the experiment also permitted excluding the possibility that *in vitro*-produced *Ss*OGT was degraded after its translation and upon the irreversible transfer of the fluoresceinated-benzyl group to the active site, as previously demonstrated [24, 25]. In effect, incubation for 60 min at 70°C of the recombinant *Ss*OGT in the *S. solfataricus* lysates in the presence of the SNAP-Vista Green™ did not affect the activity nor the fluorescent signal obtained (Figure 6, lane 3).

This analysis allowed us to estimate the amount of *in vitro*-translated *Ss*OGT to an order of magnitude corresponding to about 10–20 ng of protein produced for *μ*g of plasmid used, in 25 *μ*l of reaction.

## 5. Discussion

The present study reports the development of a transcription/translation system for the synthesis of proteins at high temperature (70°C), based on an S30 extract from the thermophilic crenarcheon *S. solfataricus*. The system makes use of an engineered classical pBS-SK plasmid, where efficient transcription is driven by a strong promoter, corresponding to the DNA region upstream from the 16S/23S rDNA gene, while translation is stimulated by the presence of a strong SD motif ahead of the start codon of the chosen gene. The reaction works at the optimal temperature of 70°C, and maximal protein synthesis is achieved after 1 h of incubation.

We tested the system with two different genes, one encoding a ribosomal protein and another encoding *Ss*OGT, an enzyme, whose activity was determined by using a fluorescent probe, as described above. The former gene had already shown to be efficiently translated in vitro from a pretranscribed mRNA [12], and served as a starting point to tune the system. Transcription/translation of the *ogt*-encoding gene allowed us to show that the protein product was active, thereby demonstrating that it was correctly folded/modified in the *in vitro* reaction. Moreover, the possibility to use fluorescent substrates of this enzyme is a clear advantage for the quantification of the gene product, making this system flexible.

An important novelty of our system with respect to previous attempts described in the literature is that it requires only endogenous components present in the cell lysate. Indeed, the only described system for protein synthesis coupled with high-temperature translation makes use of a *Thermococcus kodakaraensis* lysate, but it requires an added thermostable T7 RNA polymerase to work [26]. Our assay is therefore an economically convenient choice, since extract preparation is simple and inexpensive.

While the present work describes a promising new technology mainly for the gene expression analysis, it is not yet usable as such for the *in vitro* scale-up production of recombinant proteins. To achieve this, further experiments and improvements are needed. For instance, one may envisage the division of the reaction into two compartments, one containing the modified extract and one containing a feeding solution that includes substrates such as amino acids, ATP, and GTP, and that is renewed by continuous flow, permitting substrate replenishment and byproduct removal.

Moreover, it should be observed that extant-coupled CFPS utilize DNA in three forms: linear PCR product, linearized plasmid, and circular plasmid. The use of linear PCR products has the distinct advantage of simplicity, since it eliminates the need for time-consuming cloning steps. However, circular DNA plasmids have typically been preferred to linearized plasmids or PCR products, due to the greater susceptibility of linear DNAs to nucleolytic cleavage. Indeed, in our case, samples incubated with the linearized plasmid failed to yield the expected protein product due to degradation of the linearized plasmid in the reaction mix. The removal of nucleases, and/or the utilization of overhang extensions to cyclize PCR products, could be adopted in the future for the optimization of the system.

In conclusion, we believe that the system described here has very good potential for use in fields such as protein display technologies, interactome analysis, and understanding of the molecular mechanisms governing coupled transcription-translation in archaea.

## Figures and Tables

**Table 1:** Experimental conditions adopted for reactions with S30 *S. solfataricus.*

	*In vitro* transcription adopted from [16]	*In vitro* transcription under our conditions	Coupled *in vitro* transcription and translation	*In vitro* translation
KCl (mM)	—	10	10	10
Tris-HCl (mM)	50 (pH 8.0)	20 (pH 6.8)	20	20
Mg(OAc)_2_ (mM)	25	20	20	20
ATP (mM)	2	2	1.5	1.8
CTP (mM)	1	1	1.5	—
GTP (mM)	1	1	1.5	0.9
UTP (mM)	0.6	0.5	1.5	—
(*α*-^32^P) UTP (*μ*M)	100	100	—	—
EDTA (mM)	1	—	—	—
DTT (mM)	1	—	—	—
Total tRNA (*μ*g)	—	—	3,3	3,3
S30 (*μ*g)	100–150	100–150	100–150	100–150
T (°C)	60	70	70	70

## Data Availability

The data used to support the findings of this study are included within the article.
